# Overall survival after reirradiation of spinal metastases – independent validation of predictive models

**DOI:** 10.1186/s13014-016-0613-y

**Published:** 2016-03-08

**Authors:** Daniel Buergy, Lena Siedlitzki, Judit Boda-Heggemann, Frederik Wenz, Frank Lohr

**Affiliations:** Department of Radiation Oncology, Universitätsmedizin Mannheim, Medical Faculty Mannheim, Heidelberg University, Mannheim, Germany

**Keywords:** Palliative radiotherapy, Spinal metastases, Survival score, Reirradiation

## Abstract

**Background:**

It is unknown if survival prediction tools (SPTs) sufficiently predict survival in patients who undergo palliative reirradiation of spinal metastases. We therefore set out to clarify if SPTs can predict survival in this patient population.

**Methods:**

We retrospectively analyzed spinal reirradiations performed (*n* = 58, 52 patients, 44 included in analysis). SPTs for patients with spinal metastases were identified and compared to a general palliative score and to a dedicated SPT to estimate prognosis in palliative reirradiation independent of site (SPT-Nieder).

**Results:**

Consistently in all tests, SPT-Nieder showed best predictive performance as compared to other tools. Items associated with survival were general condition (KPS), liver metastases, and steroid use. Other factors like primary tumor site, pleural effusion, and bone metastases were not correlated with survival. We adapted an own score to the data which performed comparable to SPT-Nieder but avoids the pleural effusion item. Both scores showed good performance in identifying long-term survivors with late recurrences.

**Conclusions:**

Survival prediction in case of spinal reirradiation is possible with sufficient predictive separation. Applying SPTs in case of reirradiation helps to identify patients with good life expectancy who might benefit from dose escalation or longer treatment courses.

**Electronic supplementary material:**

The online version of this article (doi:10.1186/s13014-016-0613-y) contains supplementary material, which is available to authorized users.

## Background

Survival from most types of cancer has increased steadily during the last years in developed countries [1]. Longer survival time is associated with an increased number of patients who live long enough to experience a recurrence after an initial radiotherapy [2]. Currently around 8–20 % of patients with bone metastases receive reirradiation [3]. Although reirradiation is possible with modern techniques, it can be associated with an increased risk of side effects, depending on reirradiation site, volume, cumulative dose, fraction dose, and interval between irradiation and reirradiation [4–6]. Due to potentially debilitating side effects such as radiation myelopathy (RM) or radiation-induced sacral (plexus) neuropathy [7–9], historically only few studies of conventional reirradiation of spinal metastases have been reported [7, 10–12]. A retrospective analysis of cases treated at the Mayo Clinic between 1975 and 1992 showed that 5 out of 54 patients developed neurologic deterioration after reirradiation. Although only one of them developed the typical Brown-Sequard syndrome, the authors could not determine if the other four patients had delayed RM, or if symptoms were triggered by spinal cord compression. Median time of onset for deterioration was 7.8 months, and median survival of all patients was 4.2 months, therefore it is possible that the number would have been higher, had patients survived longer [7]. Introduction of modern techniques such as Intensity Modulated Radiation Therapy (IMRT) or Stereotactic Body Radiation Therapy (SBRT) changed the approaches to reirradiation of paraspinal tumors profoundly. Today, as reviewed by Kirkpatrick et al. [13], multiple series on reirradiation of the spinal cord have been reported with low, albeit not completely absent risk of RM. The residual risk of RM in primary SBRT or re-SBRT has been primarily attributed to higher doses per fraction [14]. On the other hand, new treatment concepts such as multiple ablative treatments in patients with oligometastatic disease warrant higher doses to obtain sufficient tumor control [15]. If patients benefit from longer, potentially more wearisome radiation courses, depends mainly on their life expectancy. It is unknown if available survival prediction tools (SPTs) can be applied to accurately predict survival in patients who are eligible for reirradiation of spinal metastases.

This study intends to validate available approaches/instruments to predict survival in a patient group who underwent reirradiation for spinal metastases in a single institution.

## Patients and methods

From November 2006 to July 2013, 58 spinal metastases were reirradiated in 52 patients. If a patient received a second reirradiation (*n* = 7), only the first reirradiation was included in further analysis. Three patients were excluded because they had received radiotherapy due to hematologic malignancies, or spinal ependymoma. Additionally, 4 patients were excluded because of missing data. The remaining patients (*n* = 44) were included in further analysis. Patient characteristics are detailed in Table 1. The study was approved by the ethics committee of Heidelberg University, Medical Faculty Mannheim (2013-602N-MA).

**Table 1:** Patient characteristics of patients who received spinal reirradiation (*n* = 44)

		Number of patients
Sex	Female	21
	Male	23
Primary tumor site	Breast	17
	Prostate	9
	Kidney (renal cell carcinoma)	6
	Lung (non-small cell lung cancer)	3
	Rectum	2
	Esophagus	2
	Head and Neck	1
	Thyroid	1
	Unknown primary	1
	Sarcoma	1
	Pancreas	1
Number of spinal metastases	*n* = 1	7
	*n* = 2	14
	*n* ≥ 3	23
Palsy	No	32
	Incomplete	8
	Complete	4
Number of extraspinal bone metastases	*n* = 0	13
	*n* = 1	13
	*n* = 2	4
	*n* ≥ 3	14
Visceral metastases	No	18
	Yes	26
	Potentially removable	6
Liver metastases	No	35
	Yes	9
Lung metastases	No	34
	Yes	10
Brain metastases	No	40
	Yes	4
Pleural effusion	No	31
	Yes	6
	Unknown (no examination documented)	7
Steroid use at time of presentation	No	26
	Yes	9
	Unknown	9
Age at start of reirradiation	Median (range)	67 (39–88)
KPS at start [end] of reirradiation	Median	70 [70]
	Range	30–100 [20–100]
Site of reirradiation [site of first irradiation]	Cervical spine	1 [3]
	Cervical and thoracic spine	5 [4]
	Thoracic spine	14 [14]
	Thoracic and lumbar spine	6 [5]
	Lumbar spine	10 [10]
	Lumbar spine and os sacrum	4 [6]
	Os sacrum	2 [2]
	Overlapping more than 2 areas	2 [0]
Period between 1^st^ irradiation and reirradiation (same site, in months)	Median	21
	Range	4-120

Survival scores were identified by a literature search using PubMed with the following search terms: Spinal [OR] Spine [AND] Metastases Survival Score. Instruments designed for specific cancer entities [16] or for patients who already had spinal cord compression [17, 18] were excluded. SPTs which evaluated other outcomes than survival [2] were also excluded. Studies detailing SPTs that did not provide a numerical score to rank patients according to risk were transformed into such a scoring system, e.g. subgroups of “favorable, moderate, and unfavorable” were transformed into numerical values to facilitate non-parametric analysis. If such transformation was not reasonably possible, SPTs were excluded [19]. In addition to spine-specific scores, we included the SPT as developed by Chow et al. [20, 21] (SPT-Chow). SPT-Chow was chosen because it is a simple and validated tool, widely applicable in palliative settings [22]. Furthermore, we included the SPT developed for reirradiation in general by Nieder et al. [23]. SPTs as developed by Tokuhashi [24], Balain [25], Bauer [26], Bollen [27], and Tomita et al. [28], were deemed appropriate. Balain et al. called their tool the *Oswestry risk index* (SPT-Oswestry). All other SPTs were named according to the 1^st^ author of the article describing the index. All details on the SPTs are summarized in Additional file 1: Table S1. Each SPT was then applied to each patient, thereby assigning all patients to prognostic groups as defined by each SPT. We used different approaches to validate each prognostic model. First, we calculated a simple index of separation (PSEP), as defined by Altman et al. [29]. Basically, PSEP is the difference between P_*worst*_, and P_*best*_, which is the difference of the probability of dying in the group with the worst prognosis, and the group with the best prognosis. As described previously [21], we calculated PSEP at 3, 6, and 12 months after start of reirradiation.

Prognostic separation was also evaluated using the D-index, as developed by Royston and Sauerbrei [30]. The D-index can be interpreted as a robust version of the hazard ratio (HR) with 1 representing the null case, i.e. a difference in predicted survival does not result in a different observed survival. In the original article, it was reported as the logarithm of the HR (log-HR; 0 representing the null case) [30]. Different D-indices were compared using Student’s t-test as described [31]. Discrimination (i.e. the ability of a model to correctly rank the patients by risk), was evaluated by calculating the Concordance-Index “C” as proposed by Harrell et al. [32]. C-index is an application of Somers’ D and gives the probability that for a randomly chosen pair of patients, the predicted and the observed outcomes are concordant. A value of 0.5 indicates no predictive discrimination (i.e. the null case), and 1.0 indicates perfect separation of patients with different outcomes [21, 32, 33]. Different C-indices were compared as described by Kang et al. [34].

Finally we assessed the impact of each item with a Cox model, calculating the (Pseudo-)R^2^-coefficient according to Cox and Snell [35]. We included only items into multivariate modeling that were correlated with survival in univariate Kaplan-Meier models with a significance level of *p* ≤ 0.05 (Mantel-Cox log-rank). All statistics were calculated using R, a language and environment for statistical computing that is available for free online [31, 36]. Survival graphs, were prepared using SPSS Version 15 (SPSS Inc., Chicago IL).

## Results

**Fig. 1:**
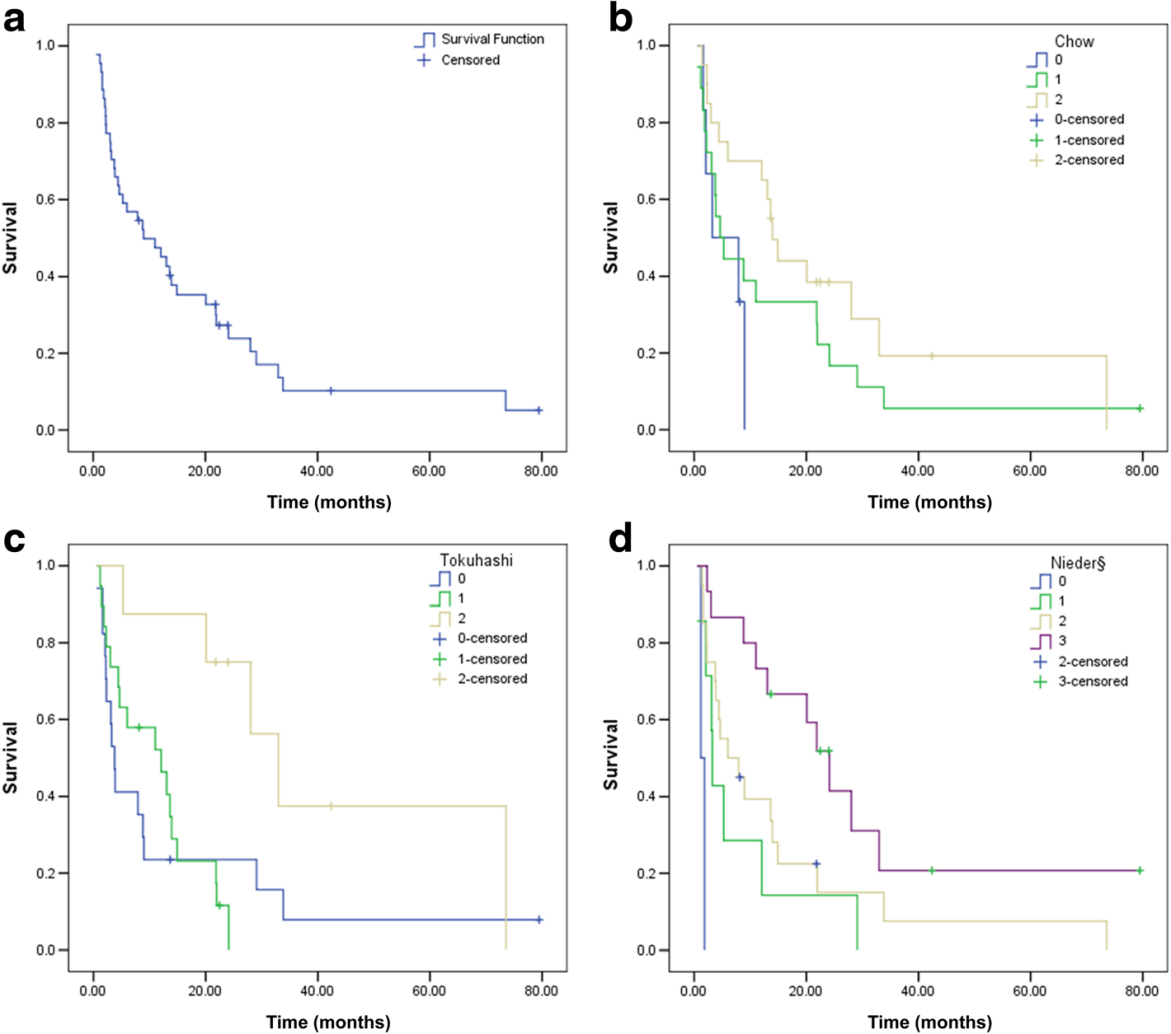
Survival curves are shown for all patients in (**a**), median OS in all patients was 9 months. **b**-**d** show survival curves in patients assigned to risk groups as detailed in each SPT that showed prognostic separation as measured by PSEP, C-index, and D-index. Median OS in the following legend is ranked from best to worst predictive group. **b** Chow, median OS: 13.9/4.6/3.2 months, *p* = 0.11. **c** Tokuhashi, median OS: 33/12.1/3.7 months, *p* = 0.033. **d** Nieder, median OS: 24.1/6/3.2/1.2 months, *p* < 0.001; § = patients with unknown PE and steroid use ranked as no PE and no steroid use

All reirradiations were applied as fractionated IMRTs. Median total dose was 30 Gy (10–45 Gy), delivered in 2 Gy fractions (range: 1.8–3.0 Gy). Reirradiation was discontinued in 4 patients due to worsening of general condition (*n* = 3) and death (*n* = 1). After a median follow-up of 36.2 months, 37 patients (84.1 %) had died. Median survival was 9 months after start of reirradiation (see Fig. 1a). Five patients (11.4 %) died within one month after their last fraction of reirradiation. We observed no case of RM or other severe late toxicities. One patient developed new sensory symptoms within 3 days after reirradiation but MRI showed no signs of RM, and symptoms resolved without intervention. Tumor control was acceptable with 9 patients (20.5 %) developing local recurrences (LR). LR occurred at a median time of 12.2 months after initiation of reirradiation. Two cases of early recurrence within 3 months were observed, both were accompanied by rapid systemic tumor progression and death within 5 months. Local control (LC) as defined by diagnostic imaging or clinical examination was maintained until death or end of follow-up in 29 patients (65.9 %). LC was undetermined in 6 patients (13.6 %), including one patient who died during reirradiation and 2 patients who discontinued reirradiation.

Patients were classified into predictive groups as described by each SPT. SPT-Nieder contains a pleural effusion (PE) item and a steroid use item (see Additional file 1: Table S1). Scores of patients with unknown pleural status were assigned to the group with no PE, i.e. no routine thorax imaging was performed when PE was not suspected. The same applies to the steroid usage item: in some patients (timing of) steroid use was not specifically documented, these patients were assigned to the group without steroid use. To avoid bias, we re-calculated all SPT-Nieder results after excluding all patients with unknown pleural status, or specific documentation of steroid use (*n* = 11). As detailed below, this did not change results significantly. PSEP showed good prognostic separation, at months 3, 6, and 12 using SPT-Nieder (73.3–86.7 %). Good late separation after 12 months was also obtained applying SPT-Chow (70 %), and SPT-Tokuhashi (64 %), however both SPTs showed weak early results in months 3 and 6. All other SPTs showed insufficient separation as measured by PSEP (see Table 2). Calculation of C-indices showed that reasonable separation of prognostic groups can be obtained using SPT-Nieder (C = 0.68, *p* < 0.001). Results were similar, if patients with unknown pleural status were excluded (C = 0.7, *p* < 0.001). SPT-Tokuhashi, SPT-Bollen, SPT-Tomita, and SPT-Chow also showed significant but numerically worse prognostic separation (C = 0.64, C = 0.63, C = 0.6, and C = 0.6, all *p* < 0.05). C-indices of SPT-Bauer and SPT-Oswestry did not differ significantly from the null case. Results obtained by calculation of D-indices demonstrated that actual risk of dying in our patient sample during follow-up was predicted by SPT-Tokuhashi (D = 0.61, *p* = 0.03, estimated HR = 1.84), SPT-Chow (D = 0.66, *p* = 0.04, estimated HR = 1.94), and SPT-Nieder (D = 1.1, *p* < 0.001; estimated HR = 3.02). All other scoring systems were not statistically different from the null case. If compared directly with the other scoring systems, SPT-Nieder showed significantly better prognostic discrimination as compared to all indices besides SPT-Tokuhashi and SPT-Chow (*p* = 0.1 and *p* = 0.11). Survival curves of all SPTs that showed acceptable results in PSEP, C-index, and D-index are shown in Fig. 1, i.e. SPT-Chow (b), SPT-Tokuhashi (c), SPT-Nieder (d). All other survival curves are shown in Additional file 2: Figure S1b-e.

**Table 2:** PSEP results after 3, 6, and 12 months

SPT	PSEP at 3 months	PSEP at 6 months	PSEP at 12 months
Bollen	15 %	15 %	35 %
Tomita	26.7 %	28.9 %	40 %
Bauer	<0 %	<0 %	4.4 %
Chow	13.3 %	25 %	70 %
Tokuhashi	35.3 %	46.3 %	64 %
Nieder^a^	86.7 %	86.7 %	73.3 %
Nieder^b^	90 %	90 %	70 %

Nieder^a^: Patients with unknown pleural status were assigned to the group without pleural effusion

Nieder^b^: Patients with unknown pleural status were excluded

If patients with unknown PE and steroid use were excluded, SPT-Nieder performed numerically better (D = 1.43, *p* < 0.001; estimated HR = 4.17) and consistently showed significantly better prognostic separation if directly compared to all other SPTs (*p* < 0.05) besides SPT-Tokuhashi (*p* = 0.06); see Additional file 2: Figure S1a for survival curve. To examine which items contributed to the performance of SPTs, we analyzed each item of each instrument in univariate survival functions. If patients had liver metastases, they died significantly earlier (*p* = 0.006, median survival 13 vs. 3.2 months). Visceral metastases in general were not significantly associated with survival. Further classification into removable vs. non-removable metastases also did not show a significant survival advantage for patients with removable vs. unremovable metastases. Karnofsky Performance Status (KPS) was significantly associated with survival (*p* < 0.001, median survival times depended on cut-off values). Steroid use was also associated with worse outcome (*p* = 0.028, median survival 13.6 vs. 3.1 months, significance was maintained after exclusion of all patients with undocumented steroid use: *p* = 0.038). All other items, including primary tumor site were not significantly associated with survival. Based on these observations, we adapted a new score to our collective. Basically this new SPT is similar to SPT-Nieder but has only three items: KPS (10–70 % vs. 80–100 %), liver metastases (yes vs. no), and steroid use (yes vs. no). In the Cox model it showed higher R^2^ (0.248 vs. 0.237; max possible 0.994 in both cases), comparison of D- and C-indices did not show significant differences as compared to SPT-Nieder (see Additional file 2: Figure S1f for survival curve).

## Discussion

In a recent survey [37], radiation oncologists reported to assess life expectancy in 91 % of their palliative patient evaluations. Their estimates were inaccurately optimistic with an overestimation of survival in 67 % of cases [37] which is in line with other physician’s survival estimates in palliative cancer care [38–40]. Predicting survival in the setting of reirradiation might be even more difficult. Patient samples are inhomogeneous, and as indicated by the historical Mayo Clinic series [7], and reproduced in our study, there is a wide range of observed survival times (historical series: 1–51 months vs. 0.5–79(+) months in our series). Except for SPT-Tokuhashi, SPTs developed to estimate survival after primary treatment of spinal metastases showed worse performance as compared to SPT-Nieder. The same was true for the well established SPT-Chow which is generally accepted as a valid survival tool in palliative cancer care [20–22]. On the other hand, SPT-Nieder which was developed in a small (*n* = 87), inhomogeneous patient sample including reirradiations for brain, bone, and lung metastases, as well as primary tumors, among others [23], showed acceptable predictive separation.

Detailed analysis of all items of the different scores, and of other patient parameters showed that SPT-Nieder included all items that were significantly associated with survival in our patient group, i.e. general condition, liver metastases, and steroid usage. Pleural effusion was the only item in SPT-Nieder that was not associated with survival in our collective. Nevertheless, we recommend to use SPT-Nieder without modification, until there is confirmation in an independent collective, that the PE item can be eliminated without loss of predictive accuracy. Consistent with Nieder’s observations, primary tumor site had no impact on survival in our patients. We emphasize that this item should be avoided as it seems to have limited accuracy in situations in which patients with aggressive tumors already lived longer than expected to experience in-field recurrence. Furthermore, new therapies, such as immunotherapeutic approaches might profoundly change prognosis in primary tumors traditionally considered to confer worse prognosis. Survival prediction is especially relevant in patients who live long enough to experience local recurrence and may require dose escalation. Interestingly, of 6 patients who developed late recurrences (11–59 months), SPT-Nieder would have ranked 4 in the best, and two in the second best (of 4) prognostic group. A potential score incorporating liver metastases, KPS, and steroid usage, which excludes the PE item would have assigned 5 patients to the long-term survivor group, and 1 patient to the second best group (of 4). All other scores ranked at least one long-term survivor in worst or second worst prognostic group. These results indicate that long-term survivors at risk for late recurrences who might benefit from dose escalation might be identified with appropriate prediction tools.

It is reassuring that despite the heterogeneous group of patients, the only survival tool which was developed for general reirradiation purposes worked best in our patient group. Our study has several shortcomings, it is a retrospective analysis in a small patient collective. Like other studies which include only patients who actually received radiotherapy, our study has an exclusion bias, i.e. patients who were not fit enough to undergo radiotherapy were not included in any analysis. This explains the low number of patients in worst prognostic groups, although many patients had late stage disease. Radiation therapy regimens in our study reflected clinical decisions involving estimated life expectancy, and other factors, but none of the SPTs were used at that time in our practice. Five patients (~11.4 %) in our study received some fractions of reirradiation during their last month of life. If radiotherapy at end of life should be completely avoided is controversial, however, single fraction or short course treatments should be preferred. A rate of 11 % irradiations during the last month of life might indicate overoptimistic clinical expectations as described previously [37].

## Conclusion

Taken together, predicting survival in patients who undergo palliative reirradiation for spinal metastases is possible with acceptable discrimination. SPT-Nieder showed best prognostic accuracy, although the pleural effusion item did not correlate with survival in our group. Radiation oncologists typically estimate survival using performance status, overall metastatic burden, presence of central nervous system metastases, and primary cancer site [37]. Our data indicate that this approach should be reconsidered when it comes to reirradiation of spinal metastases: typical predictive factors such as primary tumor, central nervous system metastases, and overall metastatic burden may have limited efficacy as compared to KPS, liver metastases, and steroid use.

## Ethics approval

The study was approved by the ethics committee of Heidelberg University, Medical Faculty Mannheim. Committees reference number: 2013-602N-MA.

## Additional files

Additional file 1: Table S1. All items that contribute to each SPT are shown and ranked according to the risk that has been assigned to each item by the authors of each SPT. (PDF 166 kb) Additional file 2: Figure S1. Survival curves in patients assigned to risk groups as detailed in SPT-Nieder (# = excluding all patients with unknown pleural status, and steroid use), SPT-Bollen, SPT-Tomita, SPT-Oswestry, and SPT-Bauer are shown in a-e. **Figure S2f.** shows performance of risk groups when ranked according to a score that was specifically adapted to the collective but has not yet been validated in an independent patient group. Median OS in the following legend is ranked from best to worst predictive group. a) Nieder, median OS: 21.8/4.6/3.1/1.2 months, *p* < 0.001; # = patients with unknown PE and unknown steroid use excluded. b) Bollen, median OS: 13/14.9/3.8/7.9 months, *p* = 0.12. c) Tomita, median OS: 20.1/2.2/7.9/3.8 months, *p* = 0.35. d) Oswestry, median OS: 14.9/6/3.1/3.8/29.1, *p* = 0.1 months. e) Bauer, median OS: 13/7.9/24.1 months, *p* = 0.75. f) Specifically adapted SPT, median OS: 24.1/8.97/3.2/1.84 months, *p* < 0.001 (TIF 4362 kb)

## Abbreviations

HR: hazard ratio
IMRT: Intensity Modulated Radiation Therapy
KPS: Karnofsky Performance Status
LC: local control
LR: local recurrence
PE: pleural effusion
PSEP: prognostic separation index
RM: radiation myelopathy
SBRT: Stereotactic Body Radiation Therapy
SPT: survival prediction tool
