# Supplementary material for: Overall survival after reirradiation of spinal metastases – independent validation of predictive models
Source: Radiat Oncol. 2016 Mar 8;11:35. doi: 10.1186/s13014-016-0613-y (PMC4782309; doi:10.1186/s13014-016-0613-y)
Supplement: Additional file 1: Table S1. — All items that contribute to each SPT are shown and ranked according to the risk that has been assigned to each item by the authors of each SPT. (PDF 166 kb) [file 13014_2016_613_MOESM1_ESM.pdf]

SPT	Item	Positive risk →					Negative risk	Risk Groups
Chow	Primary tumor site	Breast cancer	Non-breast cancer					3
	KPS	>60%	≤60%					
	Metastatic sites	No other than bones	Metastatic sites other than bones					
Tomita	Primary tumor site	Breast, prostate, thyroid, and other slow growing tumors	Kidney, uterus and other tumors with moderate growth	Lung, liver, stomach, colon, primary unknown and other fast growing tumors				3
	Visceral metastases	No	Yes. Can be treated (with local therapy)	Yes. Untreatable (with local therapy)				
	KPS	80-100%	10-70%					
Bollen	Primary tumor site	Favorable: breast, thyroid	Prostate, kidney, ovary, osteosarcoma, uterine sarcoma	Lung, colon, urothelial cell carcinoma, adenocarcinoma of unknown primary, esophagus, melanoma, pancreatobiliary, ewing sarcoma, cervix, endometrium, stomach, liver, tongue				4
	KPS	80-100%	10-70%					
	Visceral metastases	No	Yes					
Nieder	Liver metastases	No	Yes					4
	KPS	70-100%	10-60%					
	Steroid use	No	Yes					
	Pleural effusion	No	Yes					
Tokuhashi	Metastatic sites	No extraspinal bone metastases	1-2 extraspinal bone metastases	≥3 extraspinal bone metastases				3
	KPS	80-100%	50-70%	10-40%				
	Visceral metastases	No	Yes. Removable	Yes. Not removable				
	Spinal metastases	1	2	≥3				
	Primary tumor site	Thyroid, breast, prostate, carcinoid tumor	Rectum	Kidney, uterus	Others	Liver, gallbladder, unidentified	Lung, osteosarcoma, stomach, bladder, esophagus, pancreas	
	Palsy	No	Incomplete	Complete				
Oswestry	Primary tumor site	Breast, thyroid, prostate, myeloma, haemangioma, endothelioma, non-Hodgkins lymphoma	Kidney, uterus, tonsils, epipharynx, synovial cell sarcoma, metastatic thymoma	Stomach, colon, liver, melanoma, teratoma, sigmoid colon, pancreas, rectum, unknown origin	Lung			5
	KPS	80-100%	50-70%	10-40%				
Bauer	Primary tumor site	Breast, kidney, lymphoma, myeloma	Other primary sites excluding lung cancer	Lung cancer				3
	Metastatic sites	Solitary skeletal metastases	No visceral or brain metastases	Visceral or brain metastaeses				
	Pathologic fracture	No	Yes					
